# Effects of Gas Dissolution on Gas Migration during Gas Invasion in Drilling

**DOI:** 10.1021/acsomega.2c04097

**Published:** 2022-11-08

**Authors:** Haikang He, Baojiang Sun, Xiaohui Sun, Zhi-yuan Wang, Xuefeng Li

**Affiliations:** School of Petroleum Engineering, China University of Petroleum (East China), Qingdao266580, China

## Abstract

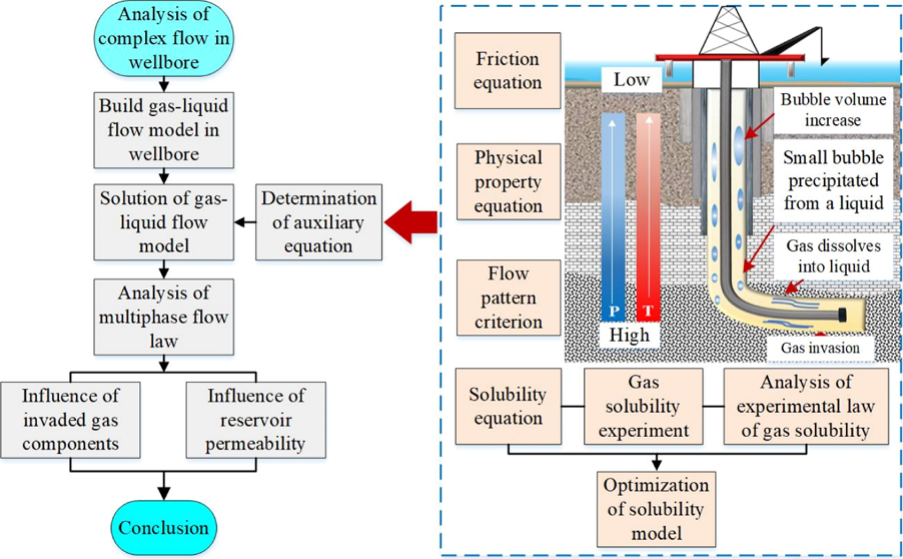

Sour gas reservoirs (including CO_2_ and H_2_S) are vulnerable to gas invasion when drilling into reservoir sections. The high solubility of the invaded gas in drilling fluid makes the gas invasion monitoring “hidden” and “sudden” for later expansion, and the blowout risk increases. Accurate prediction of gas dissolution is highly significant for monitoring gas invasion. In this study, the gas–liquid flow control equations considering gas dissolution were established. Focusing on the gas dissolution effect, a solubility experiment for CO_2_ and CH_4_ in an aqueous solution was performed using a phase equilibrium device. The experimental and simulation results revealed that the addition of CO_2_ can significantly increase gas dissolution, and the presence of salts decreases it. For solubility prediction of pure CH_4_ and CO_2_, the fugacity–activity solubility model, calculated using the Peng–Robinson equation of state, was more accurate than the Soave–Redlich–Kwong equation of state. The Soave–Redlich–Kwong equation of state has higher accuracy for the CO_2_ and CH_4_ gas mixture. If the gas dissolution effect is considered for wellbore gas–liquid flow, the time required for the mud pit gain to reach the early warning value increases. When the contents of CO_2_ and H_2_S in intrusive gases are higher, the time for mud pit gain change monitored on the ground increases, the concealment increases, and the risk of blowout increases.

## 1 Introduction

The reservoir fluid of a gas reservoir is a mixture of hydrocarbon and non-hydrocarbon gases (CO_2_ and H_2_S). There is a large number of natural gas fields containing non-hydrocarbon gases in China, including Puguang gas field in Sichuan,^1−4,43−45^ in which the content of hydrogen sulfide is 15–18% and that of carbon dioxide is 10%, belonging to the category of acid gas fields. Romania, Mexico, Argentina, and Indonesia have high carbon dioxide gas reservoirs, in which the concentration of carbon dioxide in the reservoir fluid is up to 86%,^5,6^ whereas the content of carbon dioxide in natural gas produced in the Tugu Barat oilfield in Indonesia is up to 76%.^7^ For the drilling and development of such gas fields, it is very easy to encounter abnormally high pressure and formation fluid inflow into the wellbore in fractured vuggy reservoirs. After the invasion of the formation fluid with a high content of CO_2_ and H_2_S, the high dissolution in the drilling fluid makes ground monitoring more difficult.

Considering the influence of the gas dissolution effect on the multiphase flow, scholars have conducted similar research. Thomas (1984) used Redlich – Kwong equation of state^8^ to calculate the dissolution of gas in oil-based drilling fluid. It was found that the kick increment can effectively represent the kick degree of oil-based and water-based mud.^9^ Yin et al. (2017) established an annular multiphase transient flow model considering the dissolution of gas in oil-based drilling fluid based on the gas–liquid two-phase flow and flash theory. Gas dissolution leads to a slow change in mud pit gain, and the mud pit gain of oil-based drilling fluid is less than that of water-based drilling fluid.^10^ Sun et al. (2017) established a wellbore multiphase flow model in combination with gas–liquid phase mass conversion. It was found that in the early stage of a low gas influx rate or kick, lags kick monitoring due to gas dissolution.^11^ Sun et al. (2018) considered the phase change and dissolution of acidic natural mixture in drilling fluid and proposed the flow transition criterion of multiphase flow. It focused on the gas phase analysis and did not discuss the gas dissolution effect.^12^ Xu et al. (2018) used the standing bubble point formula^41^ to calculate the solubility of gas in the oil phase. If the gas dissolution effect is ignored, the bottom hole temperature will be overestimated by 3.74 °C, and the bottom hole pressure will increase by 2.92 MPa.^13^

Research and prediction of gas solubility in water-based drilling fluids cannot be ignored. Because water-based drilling fluids are primarily composed of water and salts, Wiebe and Gaddy (1939), Briones et al. (1987), and Sabirzyanov et al. (2003) conducted a large number of experimental studies on the solubility of pure CO_2_ gas in pure water^14−16^ at temperatures above 373.15 K and pressures up to 70 MPa. However, there are few reports on the solubility of CH_4_ and CO_2_ mixtures in water and brine. Dhima et al. (1999) measured the experimental data of the solubility of the CO_2_ and CH_4_ mixture in water at 344.5 K and pressure of 10–100 MPa.^17^ Since then, Qin et al. (2008), Ghafri (2014), and Loring et al. (2017) have used different experimental methods to measure the gas–liquid equilibrium data of the CO_2_ + CH_4_ + H_2_O ternary system at temperatures and pressures of 323.15–423.15 K and 1–20 MPa, respectively. However, the accuracy of the experimental data requires further verification and analysis.^18−20,46^ Zirrahi et al. (2012) redefined the mutual parameters between gases in the equation of state of Peng and Robinson^21^ using the existing mixed gas solubility experimental data. The prediction deviation of the solubility of acid mixed gas in water is less than 5%.^22^ Ziabakhsh-Ganji and Kooi (2012) also improved the equation of state and established a gas solubility prediction model. The prediction accuracy of mixed gas is unknown.^23^ Li et al. (2014) predicted the phase equilibrium of CO_2_–CH_4_ – H_2_S brine using the fugacity–fugacity model and fugacity–activity model, and found that the fugacity–activity model has better accuracy in predicting the solubility of CO_2_ and CH_4_ mixed gases.^24^

**Figure 1:**
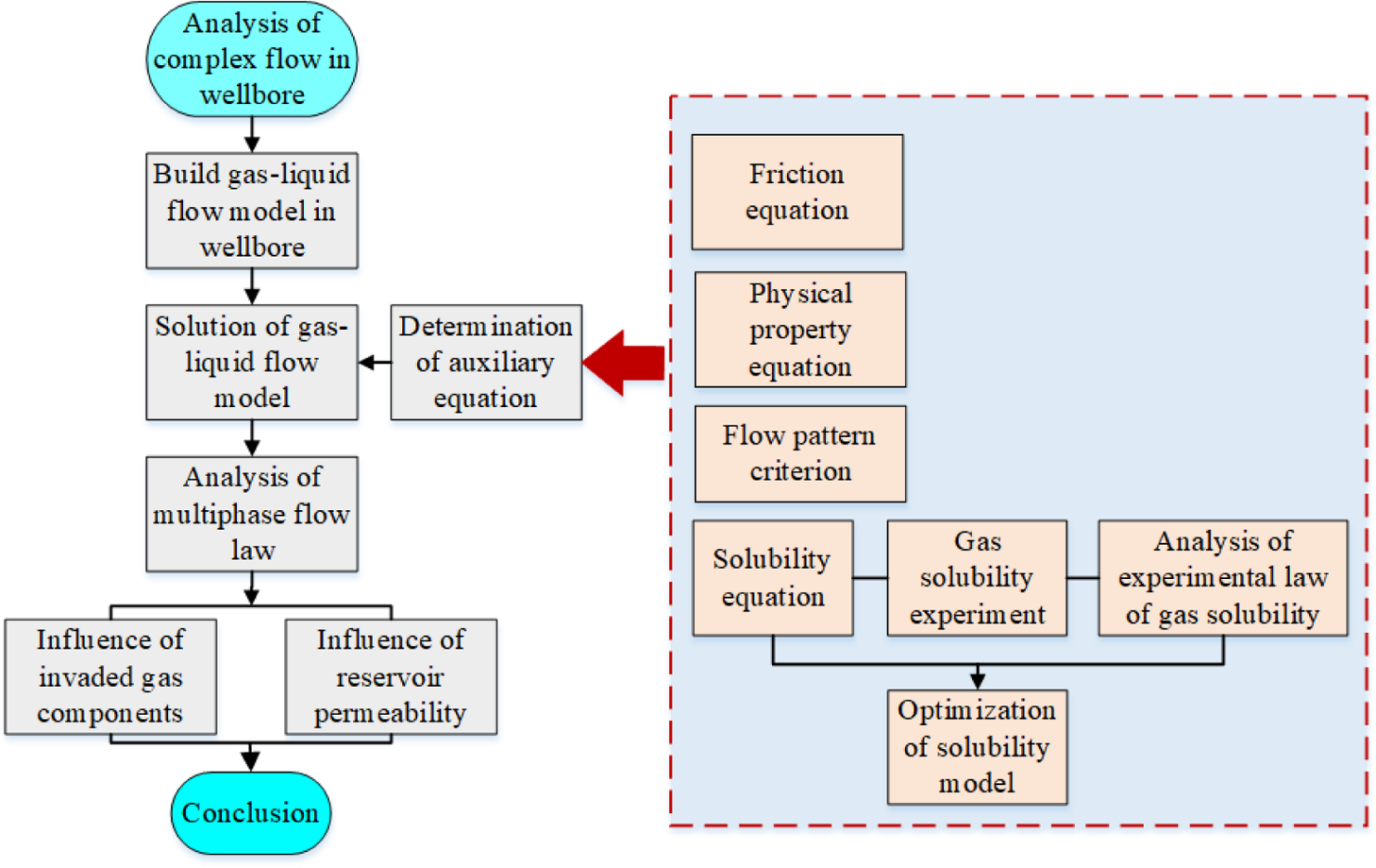
Structural guidance diagram.

Existing reports on the impact of gas dissolution on multiphase flow primarily focus on oil-based drilling fluid systems, and the low solubility of gas in water-based drilling fluids leads to neglecting these types of drilling fluid systems. Meanwhile, research on the water solubility of CO_2_ containing mixed gas focuses on the prediction of the CO_2_ and CH_4_ mixed system using the Peng and Robinson equation of state,^21^ ignoring the influence of the applicability of the equation of state itself. The structural framework of this study is shown in Figure 1. First, according to the characteristics of deep-water drilling, a gas–liquid two-phase flow control model affected by the gas dissolution effect was established. To realize the accurate prediction and analysis of gas solubility, the solubility of the CO_2_ and CH_4_ mixed gas in aqueous solution was measured using a phase equilibrium experimental device, and the solubility prediction model suitable for mixed gas was optimized. Finally, the multiphase flow model was solved, and an example was used to analyze the influence of considering gas solubility on the gas-phase flow law during gas invasion to provide guidance for the on-site well-controlled safety.

## 2 Model Establishment

**Figure 2:**
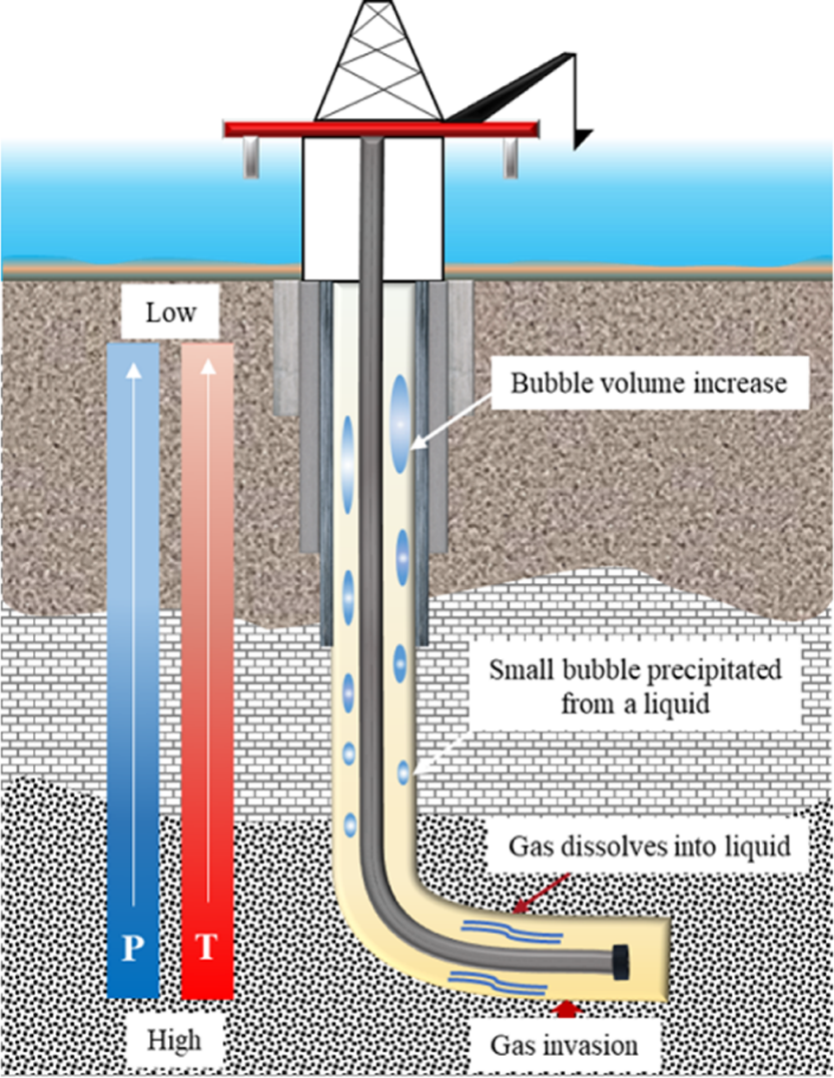
Schematic of invasive gas migration in the wellbore.

During deep-water and land drilling, the drilling into a reservoir or fractured cavernous formation is faced with the invasion of high-temperature and high-pressure gas. The migration process of the invasive gas in the wellbore is shown in Figure 2 and is divided into three stages:

(1) Formation gas invaded the wellbore. When the gas dissolution was greater than the formation gas invasion, all invaded gas dissolved into the drilling fluid and gradually migrated to the wellhead with the drilling fluid.
(2) As the dissolved gas and drilling fluid gradually migrate to the wellhead, the temperature and pressure in the wellbore decrease, resulting in a weakening of the ability of the drilling fluid to absorb gas. The corresponding amount of gas dissolution decreased and the dissolved gas precipitated in the form of small bubbles.
(3) The small bubbles in the wellbore continue to migrate upward with the drilling fluid. The wellbore temperature and pressure were further reduced and the amount of gas released gradually increased. Small bubbles gather into larger bubbles, and the gas content in the wellbore increases.

### 2.1 Gas–Liquid Two-Phase Flow Model

According to the mass conservation theorem, in addition to considering the dissolution of gas in the drilling fluid, the physical models of continuity, momentum, and energy are established based on the following assumptions:

(1) The flow in the wellbore was one-dimensional.
(2) The dissolution of gas in the drilling fluid is completed instantaneously.
(3) The change in compressibility of the drilling fluid was ignored.
(4) Ignore the influence of rock cuttings.

For the free gas phase, the continuity equation is as follows:
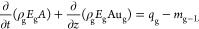
1where ρ_g_ is the density of free gas at local temperature and pressure (kg/cm^3^), *E*_g_ is the dimensionless volume fraction of free gas, *u*_g_ is the upward velocity of free gas (m/s), *A* is the cross-sectional area of the annulus (m^2^), and *q*_g_ is the mass of gas produced per unit time and thickness [kg/(s·m)]. For the nonproducing interval, *q*_g_ was 0, *m*_g–L_ is the mass transfer rate from the gas phase to the liquid phase [kg/(m·s)] and is expressed by the following equation
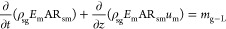
2where ρ_sg_ is the density of the standard gas under local temperature and pressure (kg/cm^3^), *E*_m_ denotes the volume fraction of the drilling fluid, *u*_m_ is the upward velocity of the drilling fluid (m/s), and *R*_sm_ is the solubility of the gas in the drilling fluid (m^3^/m^3^).

Mass conservation equation of the liquid phase is
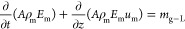
3where ρ_m_ is the density of drilling fluid under local temperature and pressure (kg/cm^3^).

Considering the slippage of the gas–liquid phase, the momentum equation of the gas–liquid phase can be expressed as
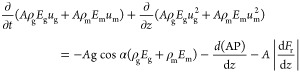
4where α is the well-deviation angle (°), *P* is the pressure (Pa), *z* is the coordinate along the flow direction (*m*), *g* represents the gravitational acceleration (m/s^2^), and d*F*_r_*/*d*z* is the frictional pressure drop of the wellbore (Pa/*m*).

There is latent heat of phase change in the gas–liquid phase equilibrium process. Considering the existence of the phase-change heat, the energy equation of the annulus in the wellbore is established as follows

Gas phase
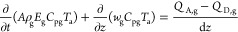
5

Liquid phase
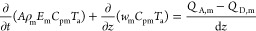
6

Change heat phase
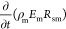
7

Therefore, the energy equation in the wellbore annulus is
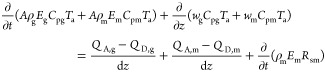
8where *T*_a_ is the annulus fluid temperature (*K*); *C*_pg_ and *C*_pm_ are the heat capacity of gas and liquid phases, respectively [*J*/(kg·K)]; *w*_g_ and *w*_m_ are the mass flow of gas and liquid phases, respectively (kg/s); *Q*_A,g_ is the heat exchange between the gas phase and annulus per unit time (*J*); *Q*_D,g_ is the heat exchange between the gas phase and drill pipe per unit time (*J*); *Q*_A,m_ is the heat exchange between the gas phase and drill pipe per unit time (*J*); and *Q*_D,m_ is the heat exchange between the gas phase and drill pipe per unit time (*J*).

### 2.2 Calculation of Frictional Pressure Drop

(1) Single phase flow

Sun et al. (2013) applied the flow of power-law fluid to the liquid-phase flow equation^25^
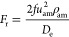
9

Re < 2000
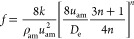
10

Re > 2000
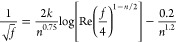
11

(2) Gas–liquid two-phase flow

Bubbly flow
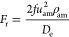
12

Slug flow and churn flow
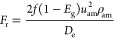
13
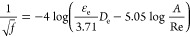
14
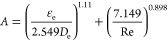
15

Annular fog flow
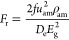
16
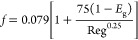
17where *u*_am_ is the average velocity of the mixed fluid (m/s), ρ_am_ is the average density of the mixed fluid (kg/m^3^), *D*_e_ is the equivalent diameter (*m*), *n* is the flow index of the mixed fluids, *f* is the friction coefficient, ε_e_ is the equivalent absolute roughness, *k* is the correction factor, and Re is the mean Reynolds number of the mixed fluids.

### 2.3 Fluid Physical Property Calculation

The Peng and Robinson equation of state (PR-EOS)^21^ was used to calculate the gas-phase compressibility factor. The basic form of the equation is as follows
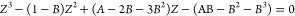
18
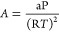
19
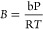
20where *R* is the general gas constant [8.314 J/(mol·K)], *P* is the corresponding pressure (MPa), *T* is the temperature (*K*), and *a* and *b* are the parameters of the gas gravity term and volume term in the PR-EOS
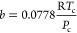
21
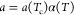
22
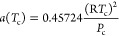
23
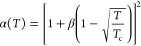
24
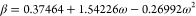
25where *P*_c_, *T*_c_, and ω are the critical pressure of component *i* in the gas (MPa), critical temperature (*K*), and the eccentricity factor, respectively (see Table 1).

**Table 1:** Critical Parameters and Eccentricity Factors of Gas Components

	CH_4_	CO_2_	H_2_S
*P*_c_ (MPa)	4.600	7.376	8.937
*T*_c_ (K)	190.6	304.2	373.2
ω	0.008	0.225	0.100

Gas density is calculated by the following equation
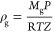
26

The viscosity calculation formula for sour natural gas in Carr et al.^26^ was adopted for calculating the viscosity of natural gases containing CO_2_ and H_2_S (as shown in Appendix A). Considering that the dissolution effect of gas requires accurate gas dissolution, the solubility prediction model will be discussed, and relevant gas water solubility experiments will be performed. For velocity and two-phase flow pattern discrimination equations, in addition to other auxiliary equations, refer to Gao et al. (2007).^27^

### 2.4 Gas Solubility Analysis

#### 2.4.1 Gas Solubility Calculation Model

The calculation of gas solubility is based on the principle of equal fugacity when the two gas–liquid phases reach equilibrium, that is,
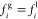
27where *f* g *i* is the fugacity of component *i* in the gas phase.
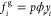
28where *f*_*i*_^l^ is the fugacity of component *i* in gas in the liquid phase, which is calculated using the following equation^47^
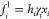
29where ϕ_*i*_ represents the fugacity coefficient of component *i* in the gas phase, γ_i_ is the activity coefficient of component *i* in the liquid phase, *x*_*i*_ is the mole fraction of component *i* in the liquid phase, *y*_*i*_ is the mole fraction of component *i* in the gas phase at gas–liquid equilibrium, and *h*_*i*_ is the Henry constant of component *i* in the liquid phase.

Therefore, the molar amount of component *i* in the liquid phase can be expressed as
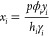
30

#### 2.4.2 Calculation of Gas Fugacity

To calculate the fugacity of component *i* in the gas phase, the PR-EOS and Soave–Redlich–Kwong equation of state (SRK-EOS) were selected. Soave (1972) considered the gravitational term in the Redlich–Kwong equation to be a temperature function in the following form^28^
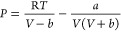
31
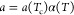
32
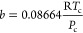
33
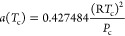
34

The improved temperature function is as follows
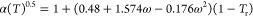
35

Therefore, the fugacity coefficient expression of component *i* is
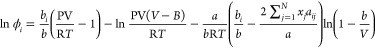
36where parameters *A* and *B* are related to temperature and pressure, respectively, and are expressed as
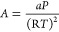
37
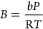
38

Peng and Robinson (1976) improved the gravitational term of the SRK equation. The basic form of the equation is as follows
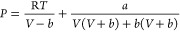
39where *R* is the general gas constant (8.314 J/(mol·K)) and *V* is the molar volume of gas (m^3^/mol). The fugacity coefficient calculation expression is
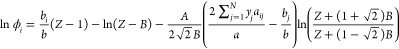
40where *b*_*i*_ is the volume parameter of component *i* and the mixture, *a*_*ij*_ is the gravitational term parameter of the mixture of components *i* and *j*, and *y*_*j*_ is the mole fraction of component *j* in the gas. The van der Waals mixing rule was adopted for the calculation of the mixed gases *a*_mix_ and *b*_mix_ by PR-EOS and SRK-EOS.
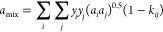
41
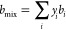
42where *y*_*i*_ is the mole fraction of component *i* in the gas phase and *k*_*ij*_ is the interaction parameter between the components. It is assumed that *k*_*ij*_ = *k*_*ji*_. The relevant parameters are listed in Table 2.

**Table 2:** Gas Intermolecular Interaction Parameters

PR-EOS				
gas	CO_2_	H_2_S	CH_4_	H_2_O
CO_2_	0	0.099^a^	0.1^a^	0.19014^c^
H_2_S	0.099^a^	0	0.084^b^	0.105^c^
CH_4_	0.1^a^	0.084^b^	0	0.47893^c^
H_2_O	0.19014^c^	0.105^c^	0.47893^c^	0
SRK-EOS				
gas	CO_2_	H_2_S	CH_4_	H_2_O
CO_2_	0	0.106^a^	0.105^a^	0.193
H_2_S	0.106^a^	0	0.0769^d^	0.105^c^
CH_4_	0.105^a^	0.0769^d^	0	0.52
H_2_O	0.193^e^	0.105^c^	0.52^e^	0

a Li and Yan.^29^

b Kontogeorgis et al.^30^

c Ziabakhsh-Ganji and Kooi.^23^

d Perez et al.^31^

e Austegard et al.^48^

#### 2.4.3 Calculation of Liquid Fugacity

The Henry coefficient method was used to calculate the fugacity of component *i* in the gas phase of an aqueous solution. For the calculation of the Henry constant of the gas component *i* in the liquid phase, the calculation equation of the aqueous solution, established by Akinfiev and Diamond, is adopted^32^
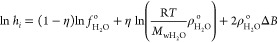
43where Δ*B* is expressed as
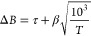
44where η is the constant of dissolved gas in water;  is the molar mass of water (g/mol);  and  represent the fugacity and density of pure water, respectively; and Fine and Millero (1973)^33^ calculated the pure water characteristics. τ (cm^3^/g) and β (cm^3^ K^0.5^/g) are adjustment calculation parameters. For CO_2_, CH_4_, and H_2_S, the above parameters were taken from Ziabakhsh-Ganji and Kooi^23^ and are listed in Table 3.

**Table 3:** Parameters and Variables Required for the Henry Constant Calculation

gas	CO_2_	H_2_S	CH_4_
η	–0.114535	0.77357854	–0.092248
τ	–5.279063	0.27049433	–5.779280
β	6.187967	0.27543436	7.262730

Calculation of activity coefficient of the gas in the liquid phase. If it is a pure aqueous solution, it is considered that the dissolution of gas component *i* in the aqueous solution is very small, λ_*i*_ = 1. In the case of the electrolyte solution, the activity coefficient of the gas component *i* adopts the calculation equation of Duan and Sun,^40^ as shown in Appendix B.

### 2.5 Optimization of the Solubility Prediction Model

To accurately analyze the applicability of the solubility model of PR-EOS and SRK-EOS used for calculating gas fugacity, the solubility of CO_2_, CH_4_, and CO_2_ + CH_4_ mixture in water was measured using a phase equilibrium experimental device. A solubility prediction model suitable for each gas component was optimized according to the experimental results.

#### 2.5.1 Gas Solubility Experiment

##### 2.5.1.1 Experimental Materials and Devices

**Figure 3:**
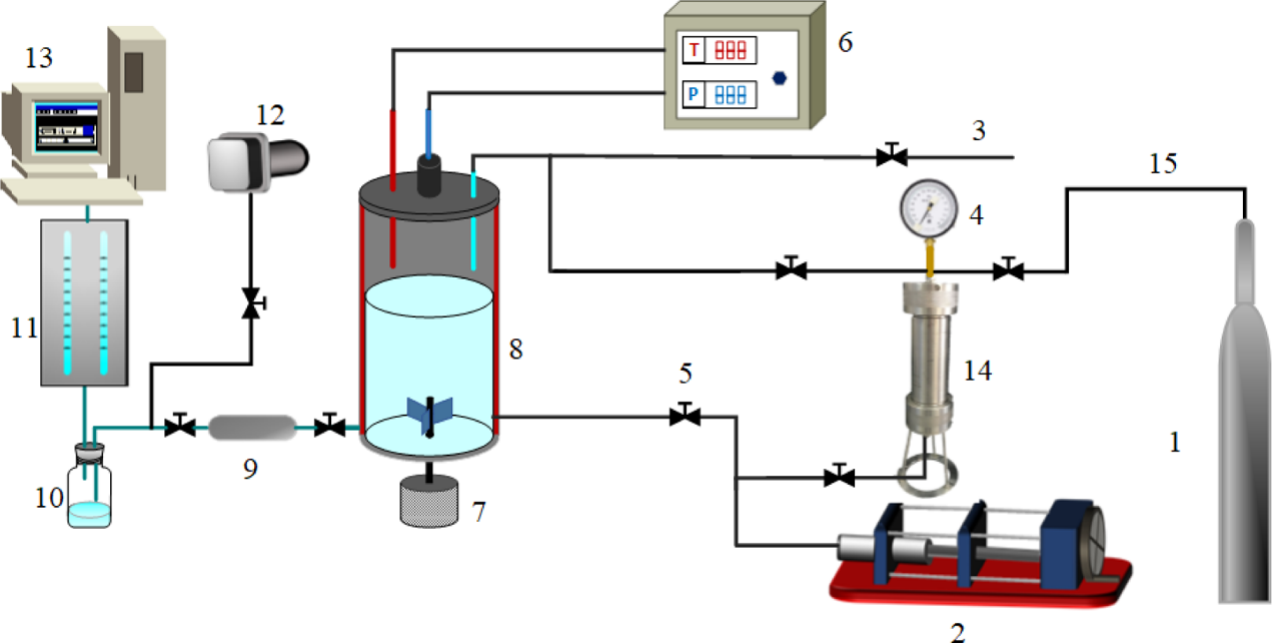
Solubility experiment experimental flow. (1) high-pressure gas cylinder; (2) constant speed and pressure pump; (3) drain pipeline; (4) pressure gauge; (5) screw valve; (6) temperature and pressure control box; (7) electromagnetic stirrer; (8) high-temperature autoclave; (9) high-pressure sampler; (10) flask; (11) gas meter; (12) vacuum pump; (13) chromatographic analyzer; (14) high-pressure intermediate vessel; and (15) pipeline.

The gas and purity used in the experiment are shown in Table 4, and Figure 3 depicts the experimental flowchart. The adopted high-temperature and high-pressure reactor had a volume of 300 mL, a maximum pressure of 60 MPa, and a maximum temperature of 473 K, and a D-250 L constant speed and pressure pump was used for pressurization.

**Table 4:** Types and Manufacturers of Gases Used in the Experiment

gas type	content	cylinder volume/*L*
CO_2_	≥99.9%	20
CH_4_	≥99.9%	20
CO_2_+CH_4_	CH_4_≥ 49.9%	8

##### 2.5.1.2 Experimental Procedures

(1) The experimental device was cleaned and its airtightness was checked. The high-temperature and high-pressure reactor was cleaned with deionized water two to three times, the intermediate container was connected to the reactor, and the reactor was boosted to 5 MPa. If the pressure was stable without fluctuation when the reactor was connected to the intermediate container within 2 h, the experimental device had good sealing; otherwise, the connection needed to be rechecked.
(2) A phase equilibrium experiment was performed by pressurization and increasing the temperature. The reactor was vacuum pumped and the liquid was injected at a constant speed using a pressure pump, and the heating device was turned on to raise the temperature to the preset temperature. A certain preset pressure of gas was filled into the reactor through the intermediate container to form a gas–liquid mixing state in the reactor. During the pressure raise process, the adiabatic system in the reactor caused certain temperature fluctuations and needed to be stabilized to the preset temperature. After stirring using an electromagnetic stirrer for 1–2 h, the observation of the pressure change in the kettle was stopped. If the pressure was stable within 3–4 h, it reached a stable state of gas–liquid equilibrium.
(3) Sample analyze, and record the data. A vacuum pump was used to vacuum the sampler to extract the liquid in the kettle, and a constant-speed and constant-pressure pump were used to inject the liquid into the reactor to keep the pressure in the kettle stable. A gas meter was used to measure the volume of the separation gas. After the gas was collected, the gas and liquid volumes were recorded in real time, measured and averaged thrice, and chromatographic analysis was conducted on the separation gas.
(4) Steps (2)–(3) were repeated to continue the solubility measurements under different pressures and temperatures.

##### 2.5.1.3 Accuracy Analysis of the Experimental Method

To verify that the above experimental device could be used for solubility measurements, the accuracy of the measured experimental data was verified. The solubility of CO_2_ gas in pure water at a temperature of 325.15 K and a pressure of 10–40 MPa was measured. The measurement results were compared with the experimental data reported by Sabirzyanov et al., Todheide and Franck, and Qin et al.,^16,18,34^ as shown in Table 5. As can be seen from Table 5, the largest average relative deviation (ARD %) of the experimental data measured in the experiment compared with the literature data is 3.54 and the smallest is 1.55, which indicates good accuracy. Therefore, the experimental device and method described above can be used for gas solubility measurements.

**Table 5:** Comparison between Experimental and Literature Values of CO_2_ Solubility in Water Measured at 323.15 K

pressure/MPa	experiment	literature value	ARD %	reference
10	0.02000	0.01868	2.63	Sabirzyanov et al.^16^
20	0.02094	0.02151	2.71	Sabirzyanov et al.^16^
20	0.02095	0.02020	3.55	Qin et al.^34^
30	0.02408	0.02494	3.54	Todheide and Franck^18^
40	0.02446	0.02484	1.55	Sabirzyanov et al.^16^

##### 2.5.1.4 Analysis of Gas Solubility Law

**Figure 4:**
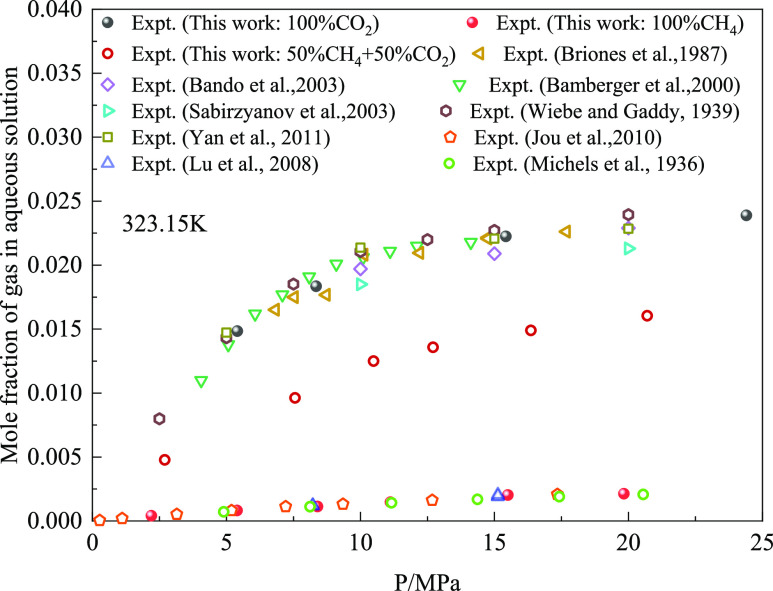
Variation of gas solubility with pressure at 323.15 K.

**Figure 5:**
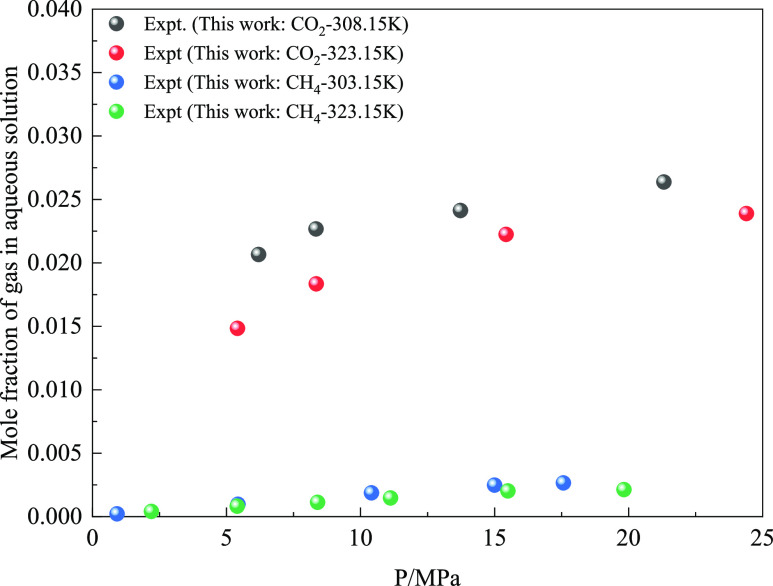
Variation of solubility of CO_2_ and CH_4_ pure gas in water with pressure at different temperatures.

**Figure 6:**
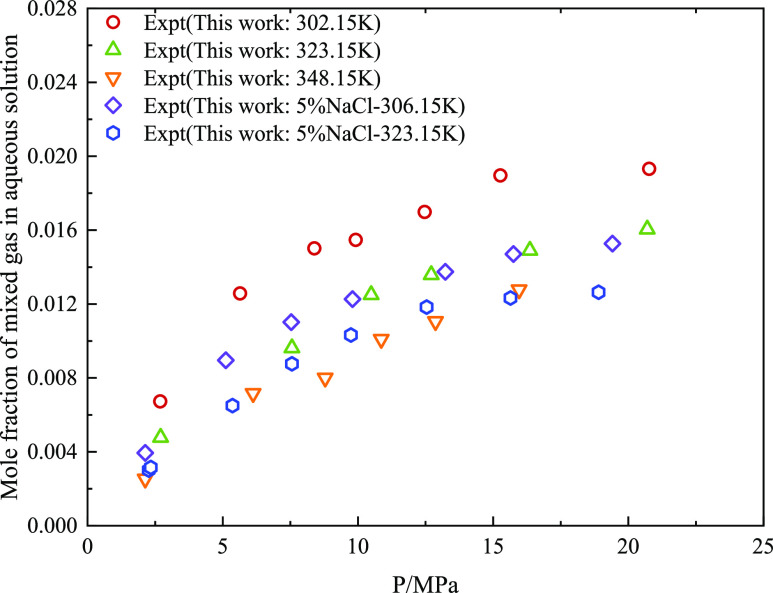
Variation of solubility of CO_2_ + CH_4_ mixed gas in aqueous solution with pressure at different temperatures.

Figures 4–6 show the relationship between the solubility of CH_4_ and CO_2_ gases in water and the pressure measured at 323.15 K. As shown in Figure 4, the solubility values of pure CH_4_ and CO_2_ measured in the experiment were consistent with those reported in the literature.^14−16,35−39,42^ Furthermore, the molar content of CO_2_ was significantly higher than that of CH_4_ gas in water. When CO_2_ gas exists in CH_4_ gas, the total dissolution of gas in water is increased. Further analysis shows that whether it is pure CO_2_ and CH_4_ or CO_2_ + CH_4_ mixed gas. When the temperature is constant, the content of gas in the aqueous solution increases gradually with the increase of pressure. However, when the pressure is constant, the solubility of the gas in the aqueous solution decreases gradually with an increase in temperature. Figure 6 shows the change in the water solubility of the CO_2_ + CH_4_ mixture when NaCl was added. When salt is added to water, the water solubility of the CO_2_ + CH_4_ mixture decreased significantly. However, its variation with temperature and pressure was the same as that of the pure gas.

#### 2.5.2 Solution of the Solubility Model

**Figure 7:**
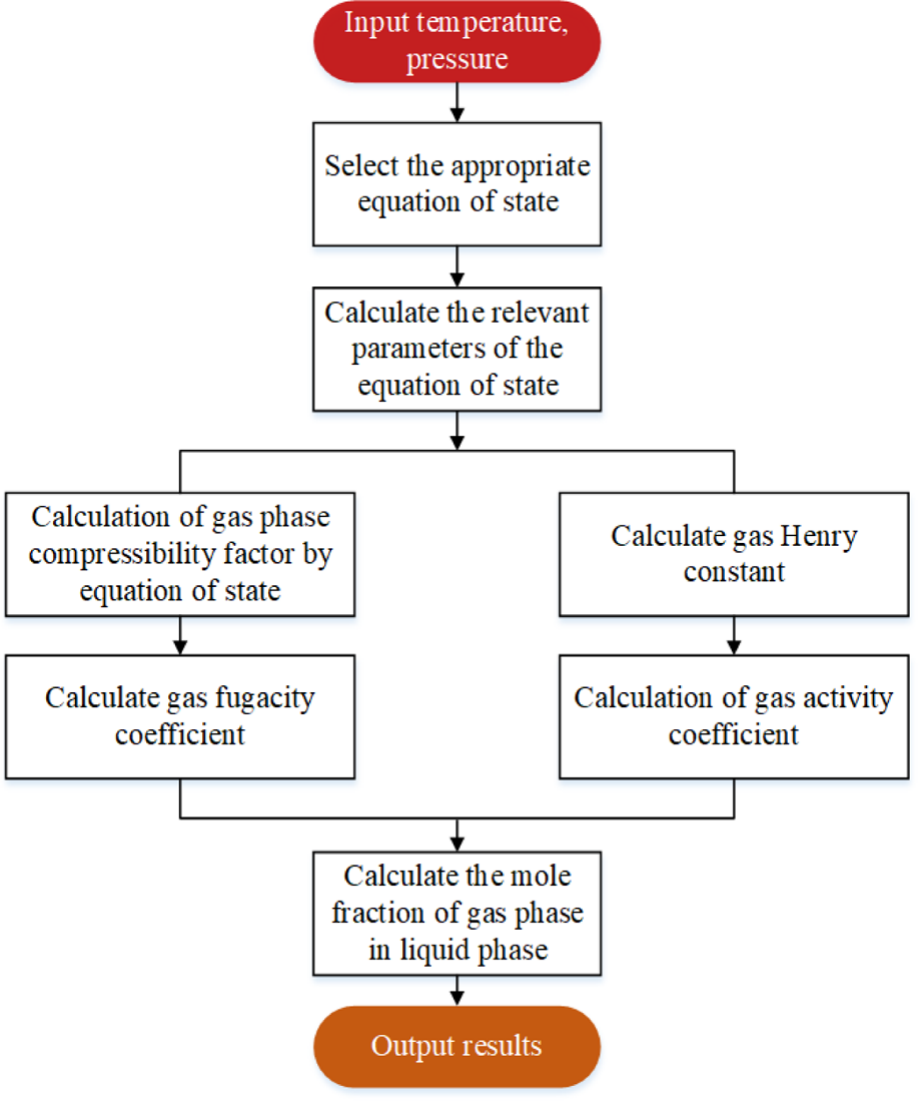
Solution flowchart of the gas solubility model.

It can be seen from eq 30 that to calculate the content *x*_*i*_ of component *i* in the liquid phase, it is necessary to know the fugacity coefficient of component *i* in gas ϕ_*i*_, Henry’s constant *h*_*i*_*,* and activity coefficient γ_*i*_. Therefore, the solubility model solution was divided into four steps (as shown in Figure 7).

1. Calculate the gas compression factor and gas mixing parameters *a*_mix_ and *b*_mix_ from the equation of state, and calculate the fugacity coefficient of component *i* in gas ϕ_*i*_*.*
2. The Henry constant *h*_*i*_ of component *i* in the gas is calculated from the Henry relation.
3. If the solution is pure water, the activity coefficient of gas component *i* is considered to be 1 and if the solution is an electrolyte solution, the activity coefficient of the gas in the liquid phase is obtained as γ_*i*_*.*
4. The calculated ϕ_*i*_, *h*_*i*_, and γ_*i*_ are substituted into eq 30 to obtain the molar content *x*_*i*_ of gas in water.

To evaluate the prediction accuracy of the gas solubility model, the average absolute relative deviation (*AARD* %) was used:
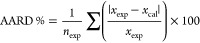
45where *x*_exp_ is the experimental data measured under experimental conditions, *x*_cal_ is the predicted value of the model, and *n*_exp_ is the number of experimental data points.

#### 2.5.3 Optimization of the Gas Water Solubility Prediction Model

For the gas solubility prediction in the auxiliary equation of the multiphase flow model, the experimental data of the solubility of pure CH_4_, CO_2_, and CH_4_ + CO_2_ mixed gas in aqueous solution were used to optimize the equation of state, and a solubility prediction model suitable for mixed gas in aqueous solution was obtained.

##### 2.5.3.1 Optimization of the Pure Gas Solubility Calculation Model

**Figure 8:**
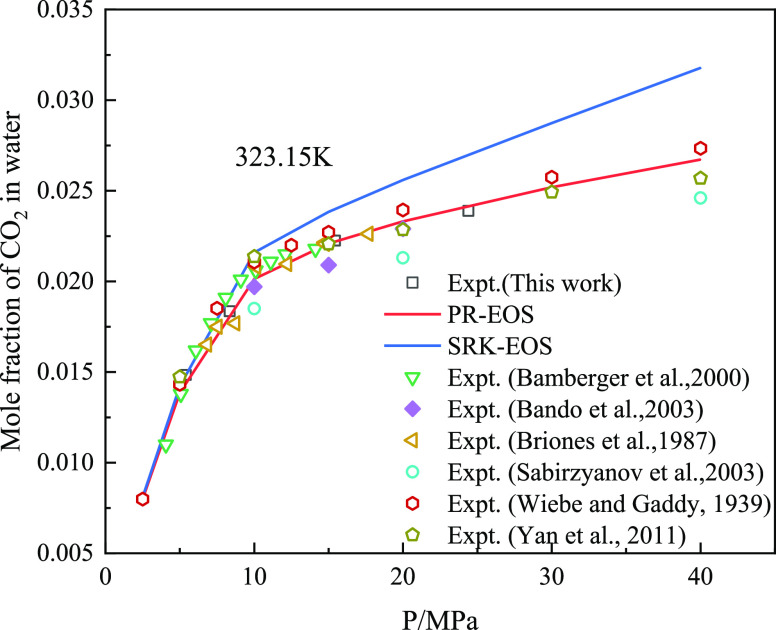
Comparison between the predicted data of the CO_2_ water solubility model and experimental data.

**Figure 9:**
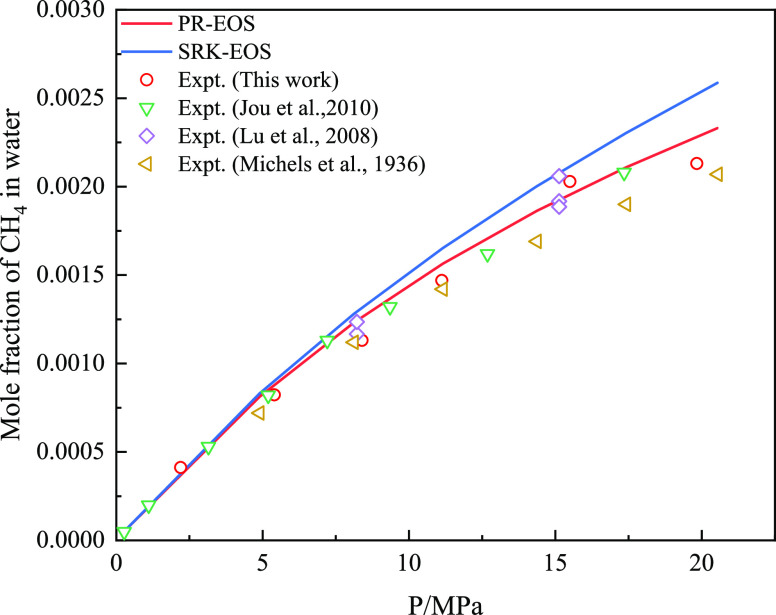
Comparison between the prediction results of the CH_4_ water solubility model and experimental data.

Solubility prediction models of different state equations were used to predict and analyze the solubilities of pure CO_2_ and CH_4_ in water, as shown in Figures 8 and 9. The AARD % calculated by PR-EOS for CH_4_ was 6.18, which was better than the AARD % calculated by SRK-EOS (9.56). For the prediction results of CO_2_ solubility in water, the AARD % values predicted by the PR-EOS and SRK-EOS were 2.98 and 8.61, respectively. Consequently, the PR-EOS has a higher accuracy in predicting the solubility of pure CH_4_ and CO_2_ in water.

##### 2.5.3.2 Optimization of the Mixed Gas Solubility Calculation Model

**Figure 10:**
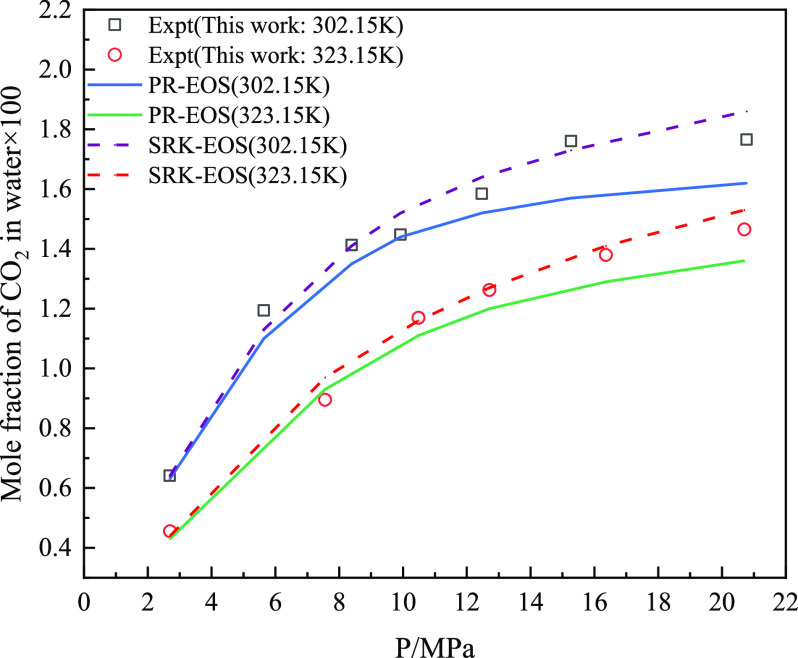
Comparison between the predicted and experimental values of the CO_2_ solubility model in aqueous solution in mixed gas.

**Figure 11:**
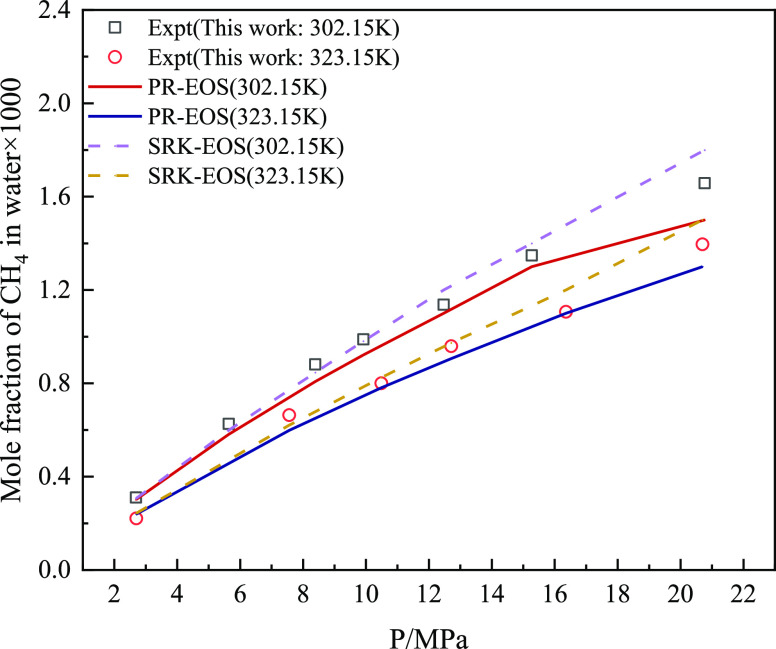
Comparison between the predicted and experimental values of the CH_4_ solubility model in aqueous solution in mixed gas.

**Figure 12:**
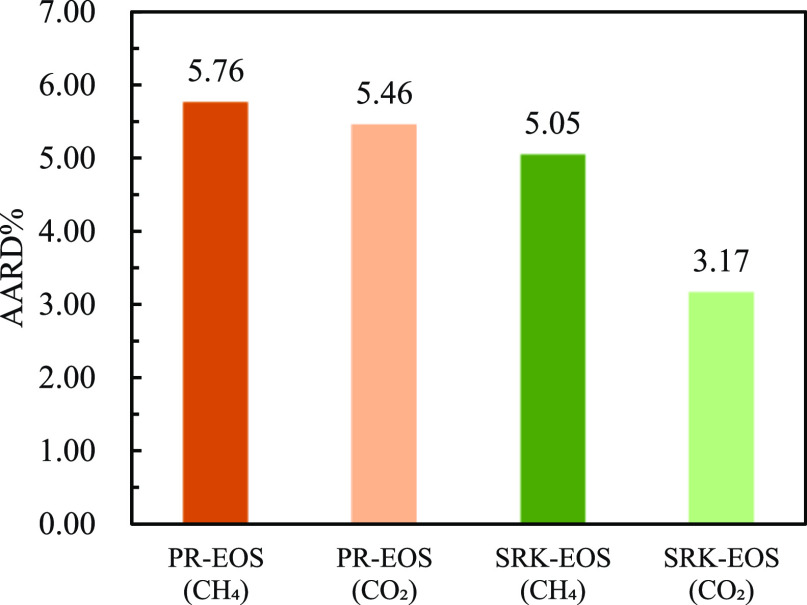
Comparison of the AARD % predicted by different equation of state models on the water solubility model of each component of mixed gas.

According to the experimental measurement results of the CO_2_ + CH_4_ mixed gas solubility, the PR-EOS and SRK-EOS models were used to predict the above experimental results (Figures 10 and 11). It can be seen from the aforementioned figures that the predicted values of the two models are consistent with the experimental values. Figure 12 shows the AARD % predicted by the two equation of the state models for the experimental results for CH_4_ and CO_2_ in the mixed gas. It can be seen that PR-EOS has the largest AARD % value for the solubility of CH_4_ and CO_2_ in water, whereas SRK-EOS has lower AARD % for the experimental results. The fugacity–activity model using SRK-EOS had higher prediction accuracy.

**Figure 13:**
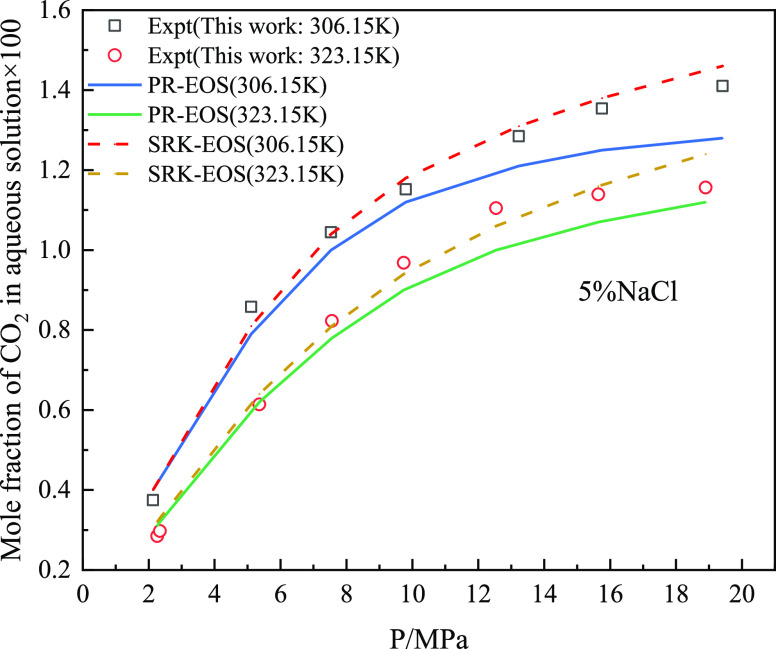
Solubility of CO_2_ in 5% sodium chloride solution in mixed gas.

**Figure 14:**
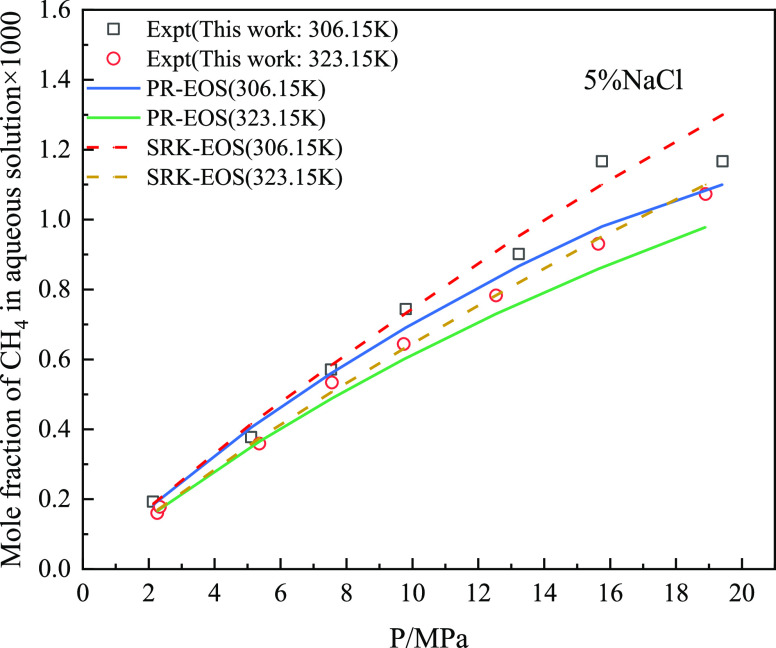
Solubility of CH_4_ in 5% sodium chloride solution in mixed gas.

**Figure 15:**
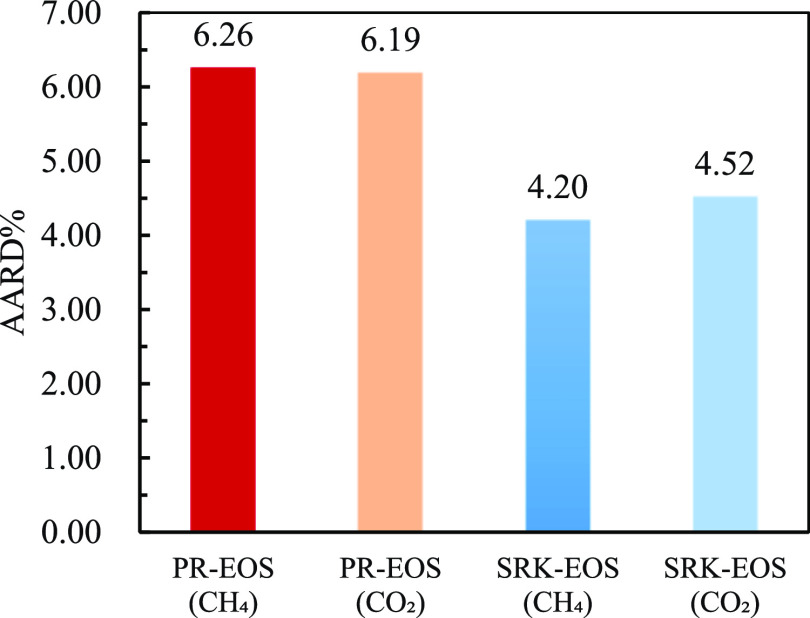
AARD % comparison results of mixed gas predicted by different models in 5% sodium chloride solution.

Figures 13 and 14 show the comparison between the experimental results and predicted value of the model for the solubility of CO_2_ and CH_4_ gas in a 5% NaCl solution in the mixed gas, and calculate and compare the predicted value of the model with the AARD % of the experimental value (as shown in Figure 15). As shown in Figure 14, AARD % calculated by SRK-EOS was lower than that calculated by PR-EOS, with an average of less than 5%. Based on the above pure gas prediction accuracy analysis, the solubility calculation model of PR-EOS was more accurate in predicting the solubility of pure gas. However, SRK-EOS was used to calculate the mixed gas composed of CO_2_ and CH_4_.

## 3 Solution of the Two-Phase Flow Model

The above multiphase flow model was processed using the finite difference method.^49^ The basic numerical discretization format is as follows
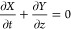
46

The basic difference format is as follows
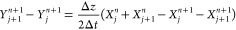
47

Discretization of the differential equation of the gas-phase no-producing interval is
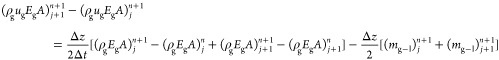
48

Discretization of differential equation of the dissolved phase is
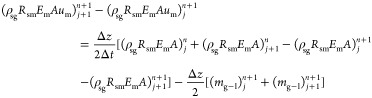
49

Dispersion of differential equation of drilling fluid is
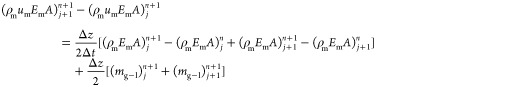
50

Discretization of momentum equation is
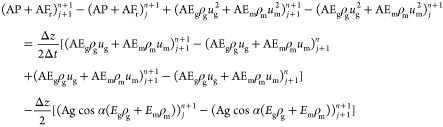
51

**Figure 16:**
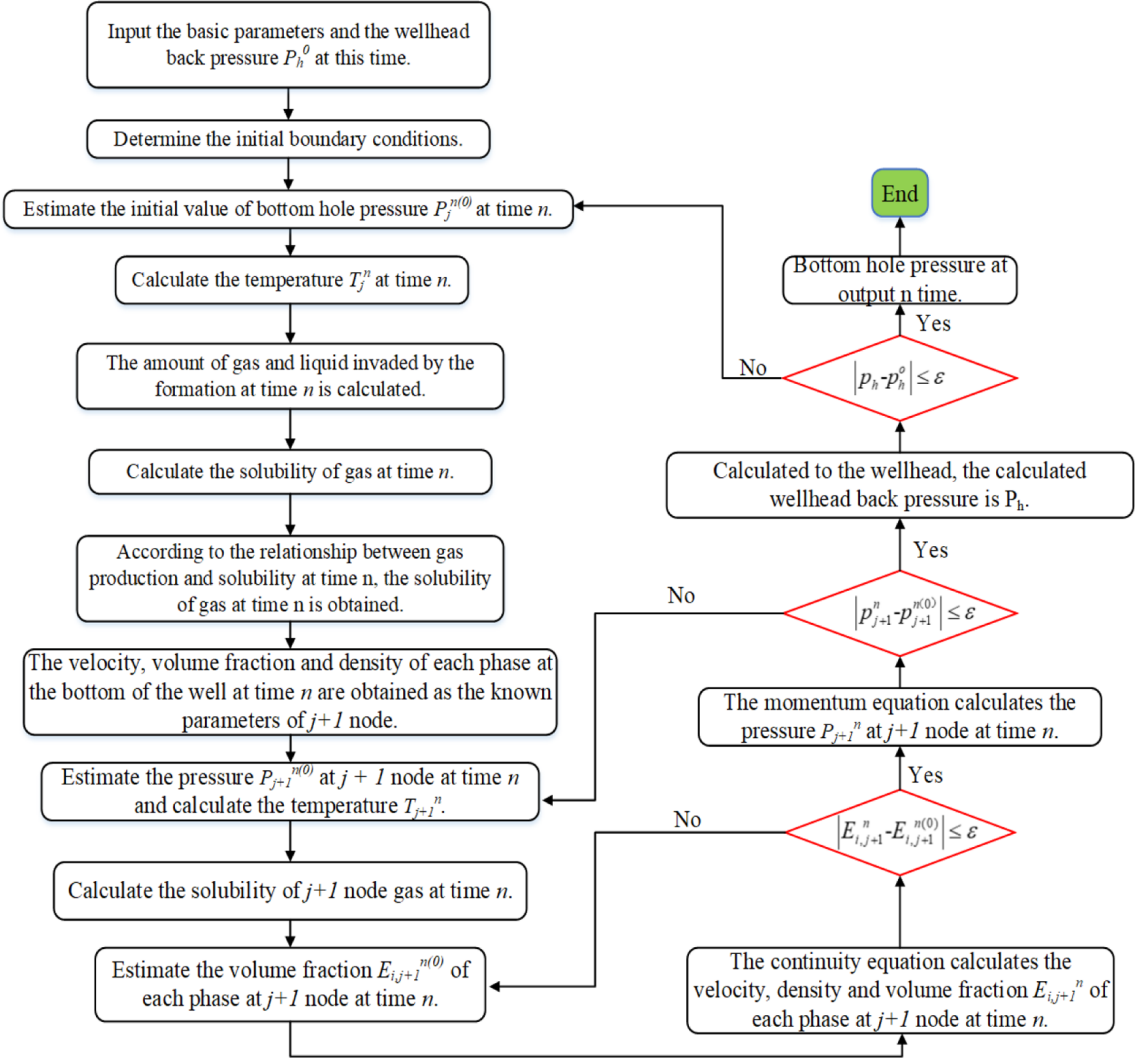
Solution flowchart of the multiphase flow model.

The solution process is illustrated in Figure 16. The specific steps of the multiphase flow model are as follows:

(1) Estimate the bottom hole pressure *p*_*j*_^*n*(0)^ at time *n* and calculate the temperature *T*_*j*_^*n*^ at time *n.*
(2) Calculate the dissolved gas of node *j* at time *n.* Based on the relationship between the calculated gas dissolution and formation gas production:
① If the calculated gas dissolution is less than the gas inflow, the gas dissolution at the current time is the calculated gas solubility.
② If the calculated gas dissolution is greater than the gas inflow, the gas dissolution at the current time is the inflow of the formation gas.
(3) According to the calculated temperature and pressure at node *j* at time *n*, output of each phase, and dissolved amount of gas, the physical property parameters of each component phase at node *j* at time *n* are calculated using the equation of state.
(4) The continuity equation was used to calculate the velocity and volume fraction  of each component phase at node *j* at time *n* as the known parameters of the *j* + *1* spatial node at time *n.*
(5) Estimate the pressure *p*_*j*+1_^*n*^ at node *j* + *1* at time *n*, repeat steps (2)–(4), and calculate the *p*_*j*+1_^*n*(0)^ of node *j* + *1* at time n using the momentum equation. If , the calculation is correct, and the parameters calculated from *j* + *1* node at time *n* are taken as the known conditions of the next *n* + *1* time; otherwise, a revaluation calculation is performed.
(6) Repeat the calculation of (2)–(5) for the wellhead; the calculated wellhead back pressure is *p*_h_. Compared with the measured wellhead back pressure *P*_h_^0^, and if , the assumption of bottom hole pressure *p*_*j*_^*n*^ at time *n* is correct. If not, return to step (2) and assign a new value to the bottom hole pressure *p*_*j*_^*n*(0)^ at time *n.*

## 4 Results and Discussion

### 4.1 Verification and Analysis of the Multiphase Flow Model

**Figure 17:**
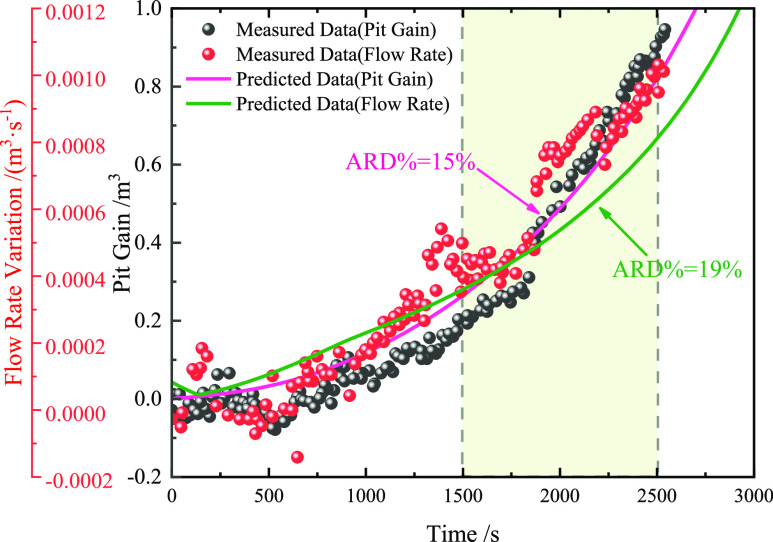
Comparison between the model predicted value and measured data [Data from Sun et al. (2007).^11^]

The measured data reported by Sun et al. (2017)^11^ are used to verify and analyze the accuracy of the multiphase flow model in this study, as shown in Figure 17. Figure 17 shows that the prediction results of the multiphase flow model for the mud pit gain increment and the flow rate were consistent with the measured data. According to the AARD % calculation of the model prediction results and the measured data in the period of 1500–2500 s, it is found that the prediction accuracy is maintained within 20%. According to Sun et al.,^11^ the measured data are more random and unevenly distributed and are significantly affected by environmental factors (sea breeze and equipment accuracy).

### 4.2 Analysis of Multiphase Flow Law

To deeply explore the influence of gas dissolution on the multiphase flow law, specific engineering example data were used for analysis, as shown in Table 6.

**Table 6:** Basic Data of Simulation Example

reservoir temperature/°C	125	reservoir pressure/MPa	53
length of gas invasion section/m	5	porosity/%	12
permeability/md	10	compressibility/(1·MPa^–1^)	2.0 × 10^–4^
temperature gradient/(° C·(100 m)^−1^)	3	drilling rate/(m·h^–1^)	10
gas invasion type	75%CH_4_ + 25%CO_2_	gas invasion time/s	2100
water depth/m	870	well depth/m	4934.4
gas invasion point/m	3857	vertical depth/m	3857
mud density/(kg·m^–3^)	1280	displacement/(L·s^–1^)	33
seawater temperature/°C	15	mud viscosity/cP	30

**Figure 18:**
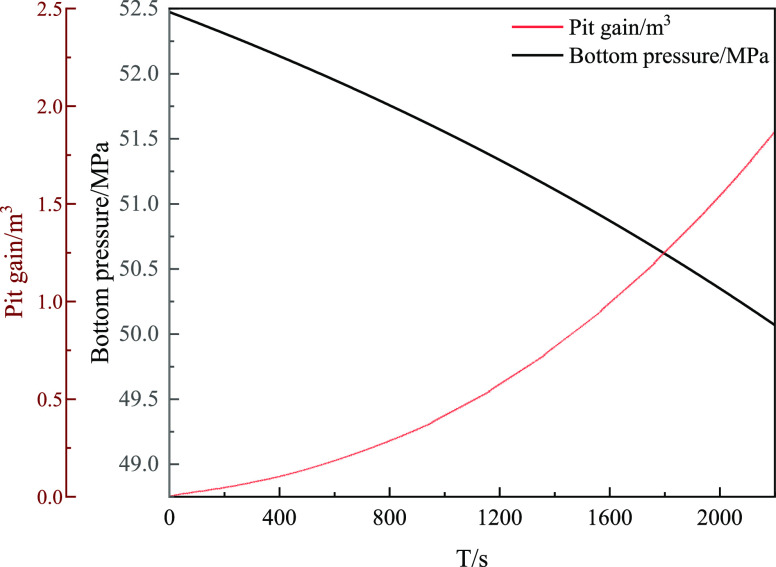
Relationship between mud pit gaint and bottom hole pressure with invasion time.

**Figure 19:**
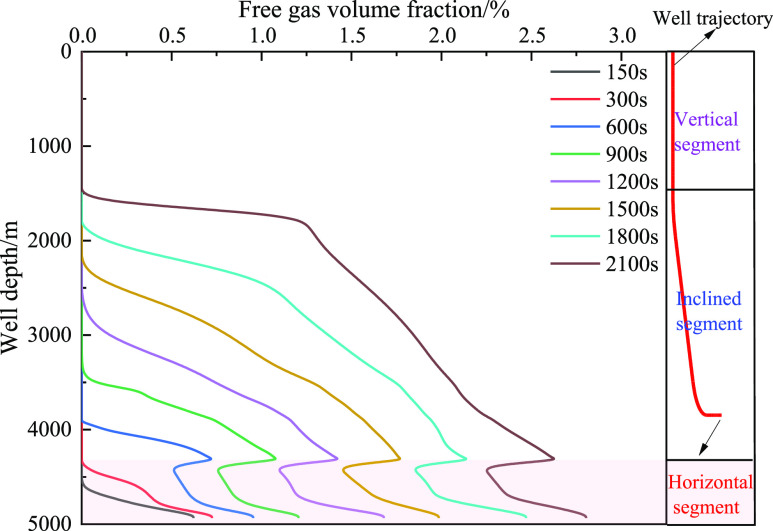
Variation of the free gas integral number with well depth at different times when gas influx is 2 m^3^.

**Figure 20:**
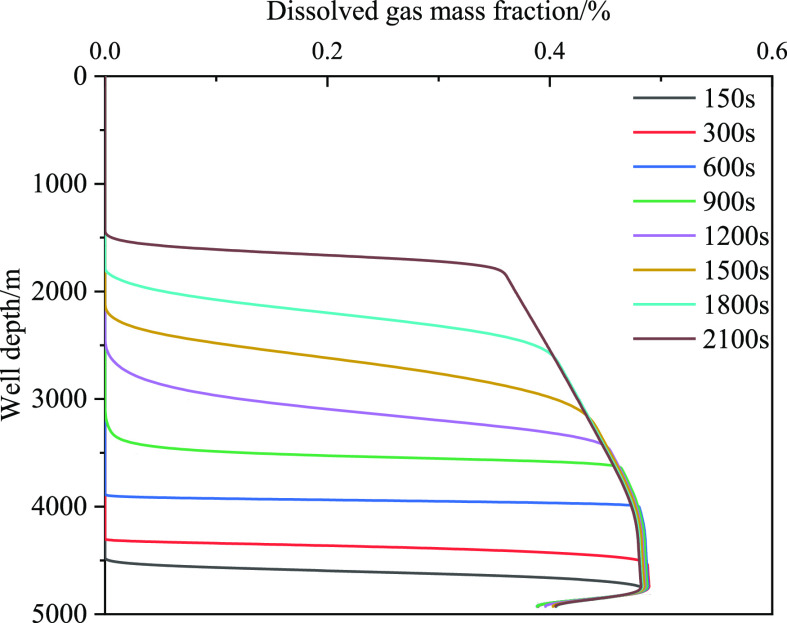
Variation of the integral number of dissolved gas with well depth at different times when gas influx is 2 m^3^.

**Figure 21:**
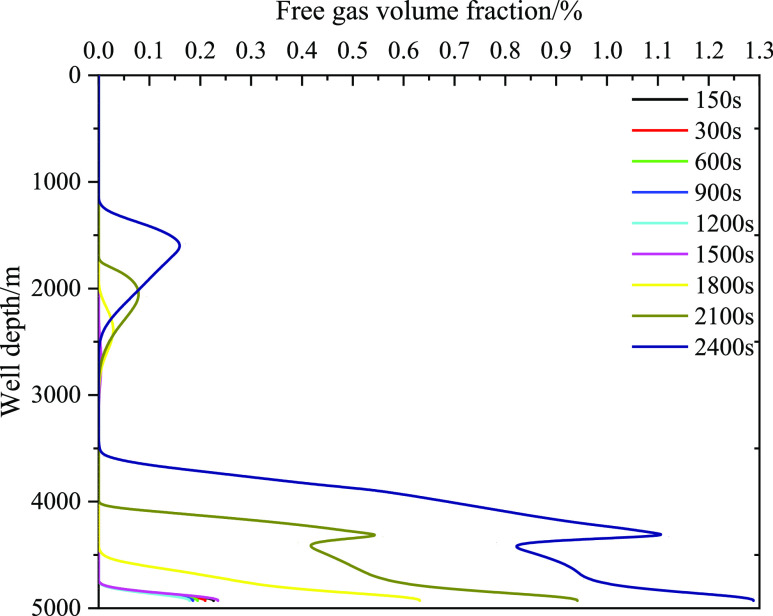
Variation of the integral number of dissolved gas with well depth at different times when gas influx is 0.9 m^3^.

**Figure 22:**
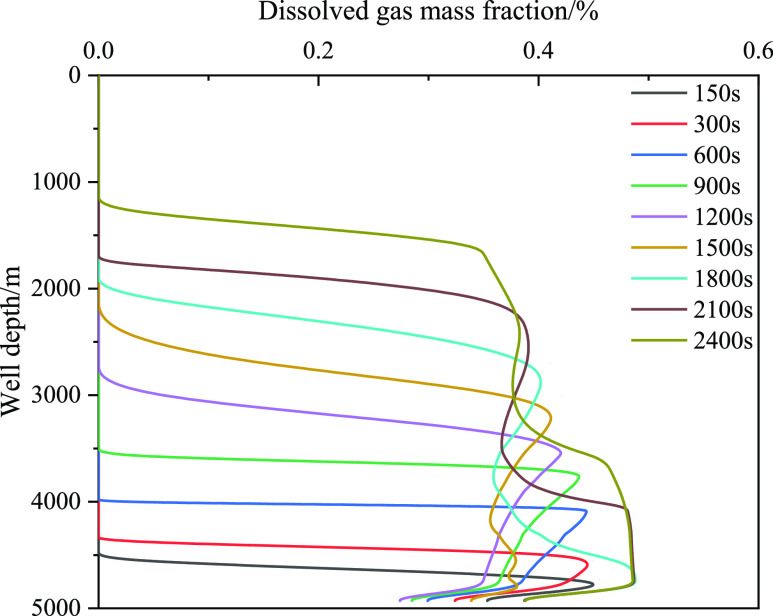
Variation of the integral number of dissolved gas with well depth at different times when gas influx is 0.9 m^3^.

Figure 18 shows the relationship between the mud pit gain and bottom hole pressure with the invasion time when the total invasion volume was 2 m^3^. The bottom hole pressure shows a downward trend with the increase in invasion time, which is mainly due to the increase in gas invasion volume owing to the gradual dissolution of the invaded gas in the drilling fluid in the horizontal section, increase in free gas volume, and decrease in friction. Figures 19 and 20 show the relationship between the fraction of free gas and dissolved gas in the annulus and well depth, respectively. As the well depth decreased, the mass fraction of the dissolved gas gradually decreased, and the corresponding integral number of free gas gradually decreased. The main reason is that the amount of gas invaded by the formation is limited, the gas gradually extends forward, and gas dissolution leads to a decrease in the volume fraction of the front gas. However, when the well depth is certain, such as 4000 m, the volume fraction of free gas increases with the increase in invasion time, which is primarily caused by the limited dissolution. Figure 20 shows that the dissolved gas content in the wellbore has a certain limit, and the dissolved gas content does not increase after exceeding the liquid phase saturation value. To further study the influence of gas dissolution, the changes in the free and dissolved gas volume fractions in the wellbore under a low gas influx (0.9 m^3^) were analyzed (Figures 21 and 22). Figure 21 displays the integral number of free gas in the wellbore before 1800 s, in addition to the increasing trend at the bottom of the well. There is no free gas in the annulus, and the corresponding volume fraction of dissolved gas increases, indicating that the gas dissolved in the drilling fluid has not reached the saturation state. At 1800 s, free gas precipitated near the wellhead, and the closer it was to the wellhead, the more the free gas content increased, mainly because of the small amount of dissolved gas near the wellhead. When the gas dissolved and reached saturation in the drilling fluid, the dissolved gas began to release, causing gas volume expansion. Meanwhile, the closer is the expansion position to the wellhead, thereby increasing the risk significantly.

### 4.3 Analysis of Influencing Factors of Flow Law

#### 4.3.1 Sensitivity Analysis 1: Influence of Different CO_2_ Content

**Figure 23:**
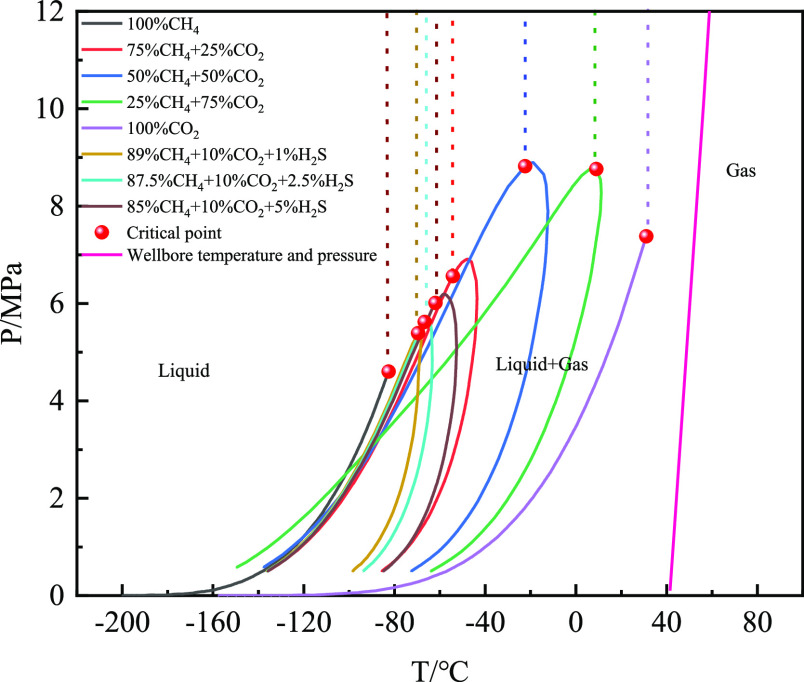
P-T phase distribution under different gas types.

**Figure 24:**
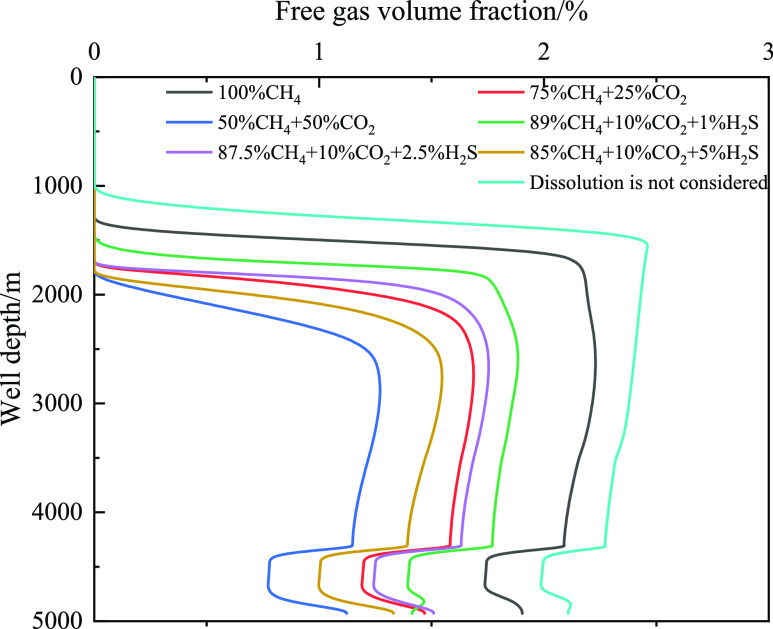
Variation of the dissolved gas integral number with well depth for different types of invasive gas.

**Figure 25:**
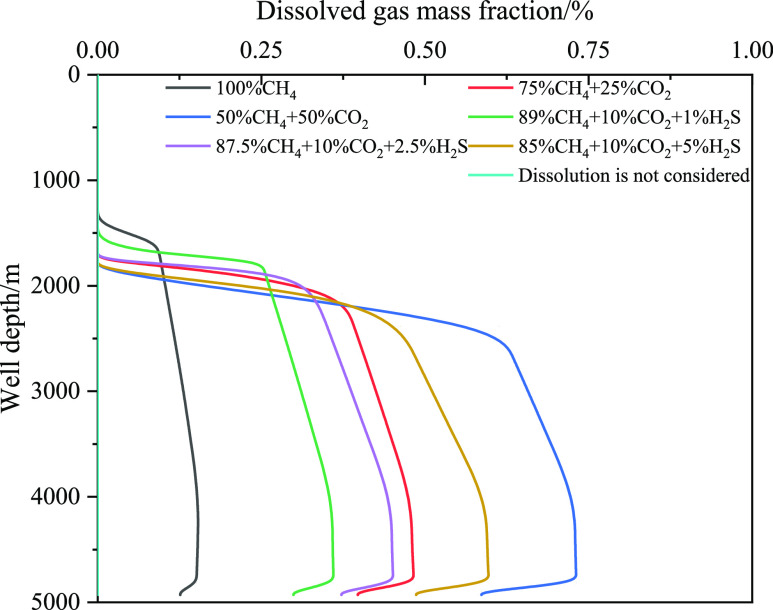
Variation of the dissolved gas integral number with well depth at different times for different types of invasive gas.

The gas–liquid two-phase flow under the conditions of constant gas influx of 2 m^3^, gas influx time of 30 min, and displacement of 2000 L/min was analyzed. Figure 23 shows the P-T phase distributions of the different gas types. It can be observed that the wellbore temperature and pressure lines are primarily located in the gaseous area. Therefore, the invaded gas is mainly in the gas phase in the wellbore. Figures 24 and 25 show the variation in the free gas volume and dissolved gas mass fractions with well depth for different acid gas contents. With an increase in the CO_2_ content, the free gas decreased and the corresponding dissolved gas content increased. The increase in acid gas content leads to a sharp increase in gas dissolution in the drilling fluid, and more gas enters the drilling fluid, causing the free gas to decrease. With an increase in H_2_S content, the change trend of the free gas volume and dissolved gas mass fractions was the same as that of CO_2_. Moreover, if the influence of gas solubility is not considered, the free gas content in the wellbore is the largest, and the corresponding gas velocity is also the largest (as shown in Figure 25).

**Figure 26:**
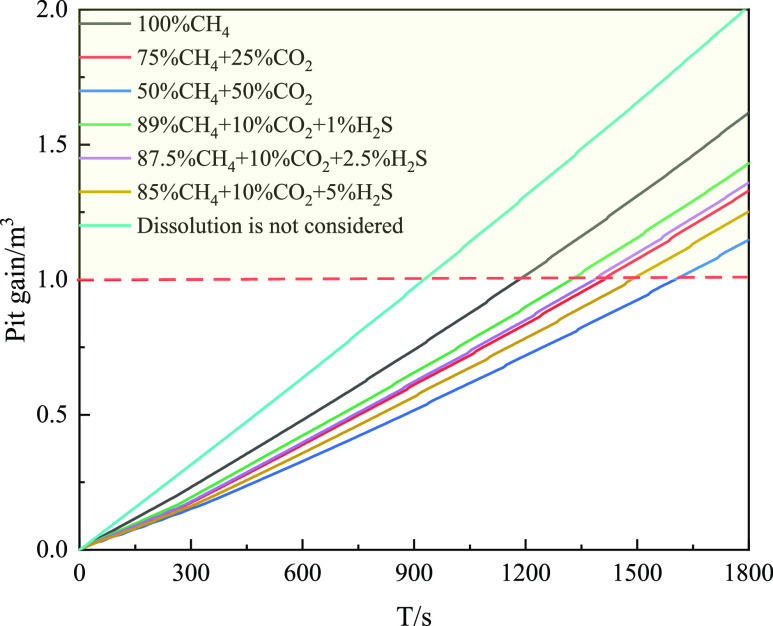
Relationship of mud pit gain with time in different gas invasion types.

Figure 26 shows the variation in the mud pit gain with the gas invasion time under different acid gas contents. If the influence of gas solubility is not considered, the gas invasion monitoring time is shorter. When the invaded gas contains high concentrations of CO_2_ and H_2_S, the incremental change time of the mud pit increases. If 1 m^3^ is taken as the monitoring value of mud pit gain, 424 s can be monitored without considering gas dissolution, 710 s is required when only CH_4_ gas dissolution is considered, and 980 s is required for intrusive gas containing 25% CO_2_. Consequently, the dissolution of acid gas leads to a certain lag and an increased risk of gas invasion monitoring.

**Figure 27:**
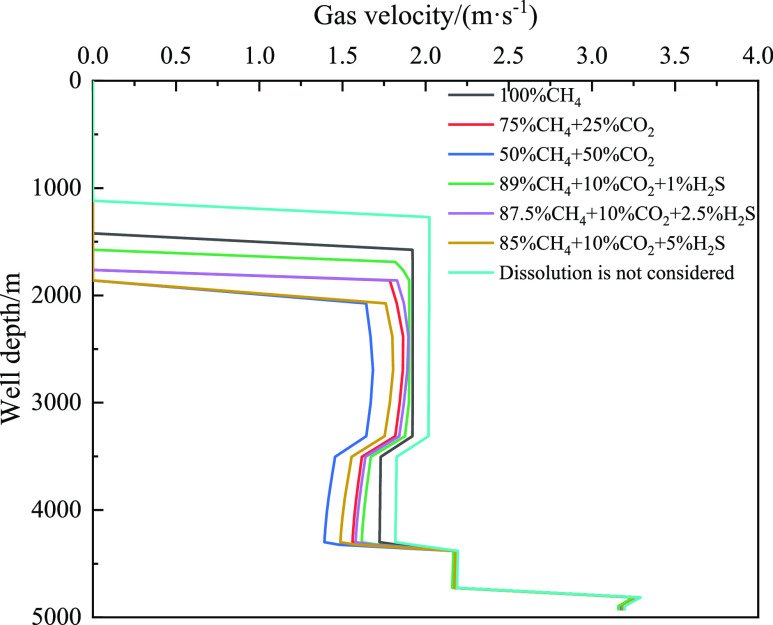
Variation of the dissolved gas integral number with well depth at different times for different types of invasive gas.

**Figure 28:**
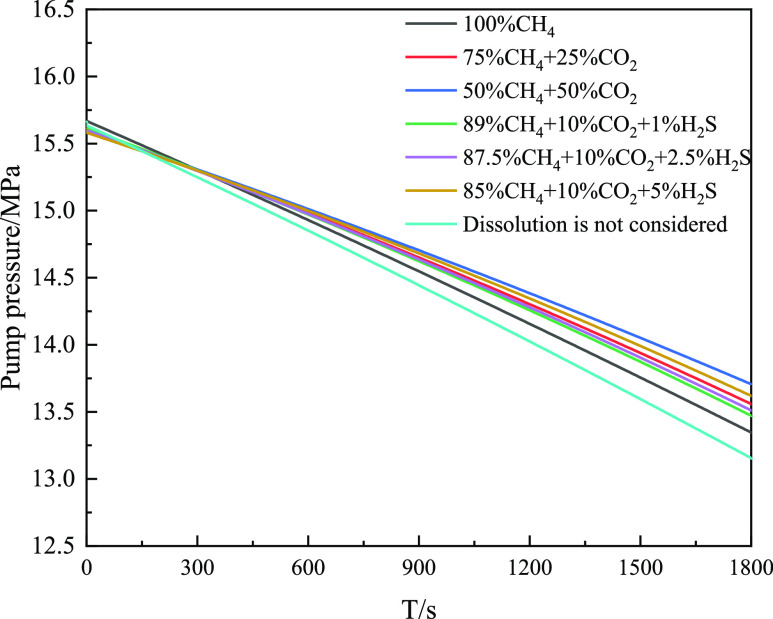
Variation of pump pressure with time for different types of invasive gas.

Figure 27 shows the distribution of the gas velocity with well depth for different acid gas contents. With an increase in the CO_2_ content of the invaded gas, the velocity of the gas in the wellbore decreases. In addition, the farther the gas front is from the wellhead. Similarly, with the increase in H_2_S content in the invaded gas, the variation law of CO_2_ is the same. It was also discovered that without considering the gas dissolution effect, the gas migration velocity was the largest, and the closer the gas front was to the wellhead. Figure 28 reveals that the pump pressure gradually decreases with an increase in the invasion time. When the invasion time was 1800 s, the pump pressure increased gradually with increasing CO_2_ and H_2_S contents. When cyclic drilling for 1800 s, the pump pressure considering CH_4_ gas dissolution is 0.17 MPa higher than that without CH_4_ gas dissolution. The higher the concentrations of CO_2_ and H_2_S in the intrusive gas, the greater is the difference between the pump pressure and the pump pressure without considering dissolution.

#### 4.3.2 Sensitivity Analysis 2: Influence of Different Permeability

**Figure 29:**
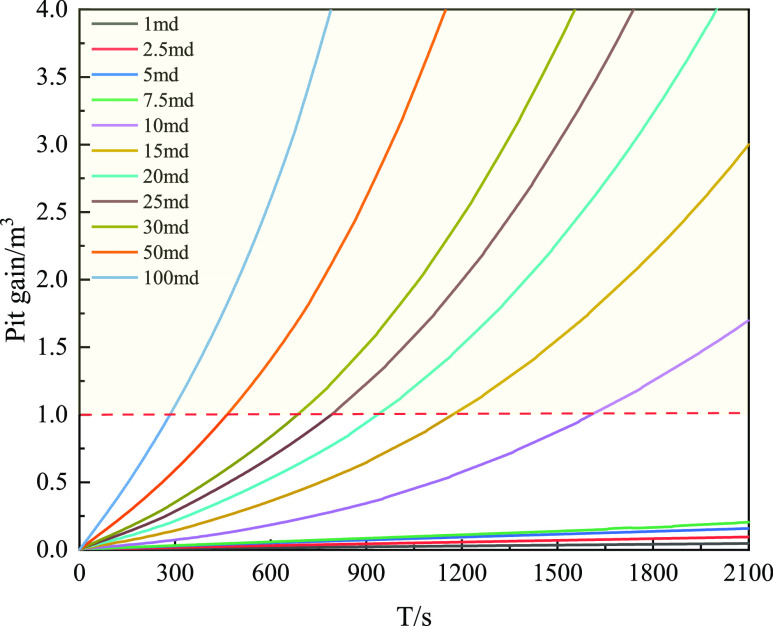
Variation of mud pit gain with invasion time under different permeabilities.

The gas–liquid flow was analyzed under the conditions: gas type 75% CH_4_ + 25% CO_2_, permeability 1–100 md, gas invasion time 1800 s, and displacement 2000 L/min. Figure 29 shows the relationship between the mud pit gain under different permeabilities and invasion times. With increasing invasion time, the mud pit gain under high permeability changes rapidly. If 1 m^3^ was used as the monitoring value, the time required to reach the monitoring value under high permeability would be longer. The main reason for this is that the increase in gas invasion under high permeability leads to an increase in gas content in the wellbore.

**Figure 30:**
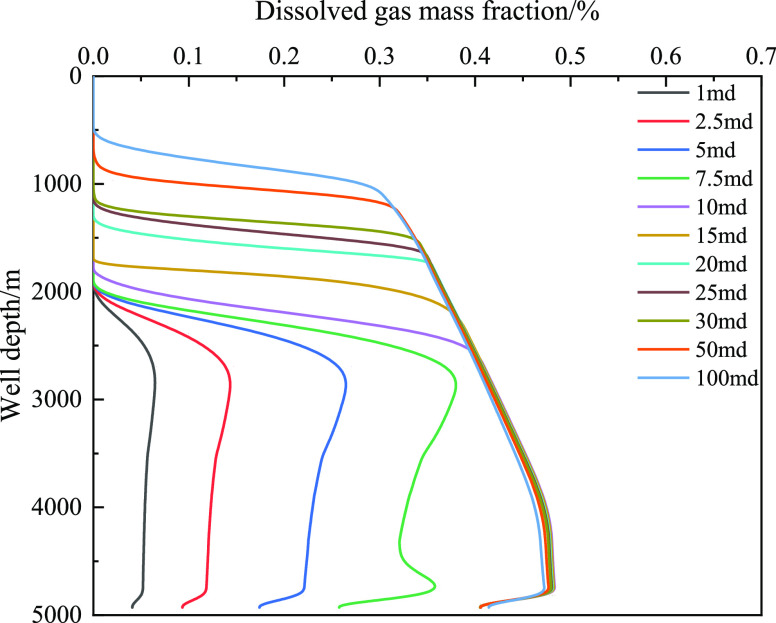
Variation of the dissolved gas integral number with well depth under different permeabilities.

**Figure 31:**
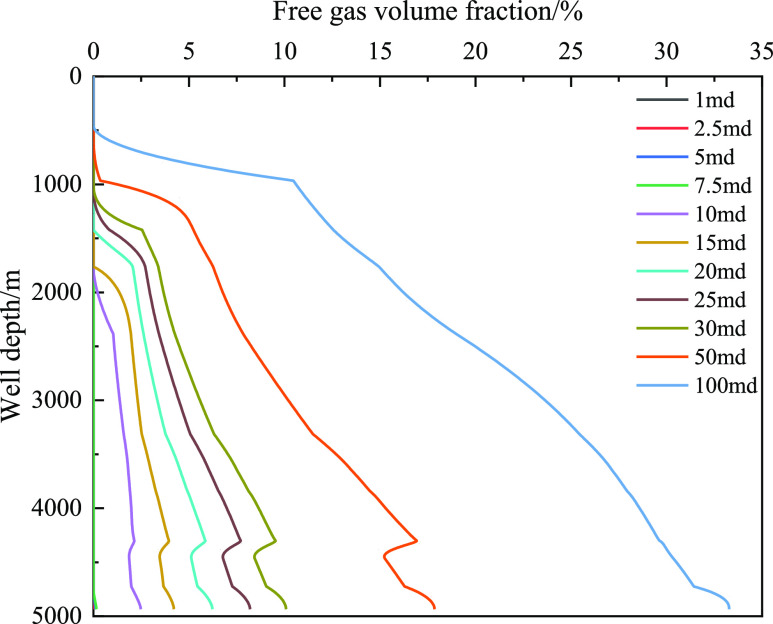
Variation of the free gas integral number with well depth under different permeabilities.

**Figure 32:**
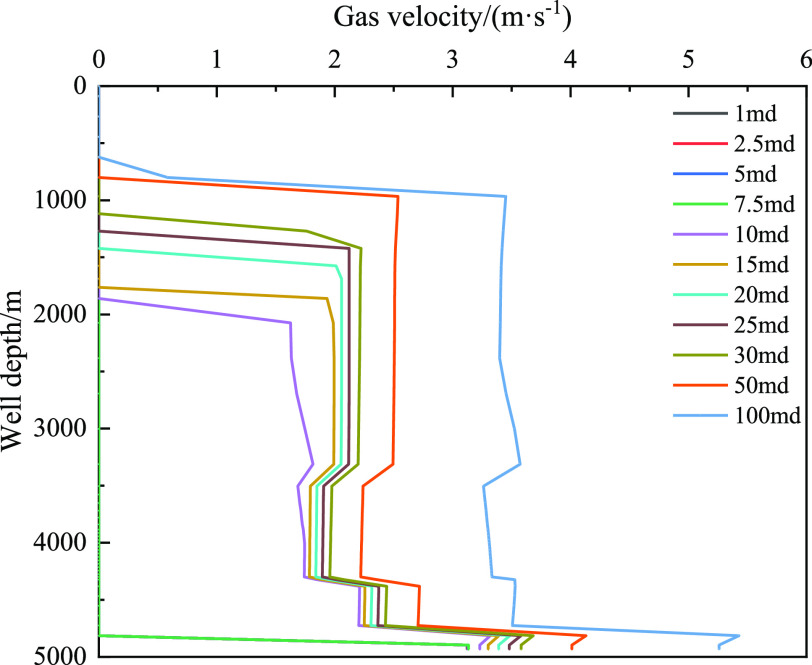
Variation of gas velocity with well depth under different permeabilities.

Figures 30 and 31 show the relationship between the mass fraction of dissolved gas and the volume fraction of free gas with well depth under different permeabilities. Under the low permeability of 1 md and 7.5 md, the amount of invaded gas is small, and the gas is completely dissolved into the drilling fluid, resulting in an integral number of free gas of 0 (as shown in Figure 30). With an increase in the permeability, the amount of gas invading the wellbore increases. Owing to the limited solubility of gas in the drilling fluid, the integral number of free gases gradually increases with an increase in permeability. A higher free gas content in the wellbore corresponds to a higher gas velocity (Figure 32), and the closer the gas movement front is to the wellhead. Hence, the dissolution of gas makes it difficult to determine gas invasion under low permeability over time, and the concealment is enhanced.

## Conclusions

Based on the dynamic analysis of the migration process of acid gas invading the wellbore, a multiphase flow model considering gas dissolution after the invasion of a highly acidic gas (CO_2_ and H_2_S) was established. The performed discussion and analysis led to the following conclusions:

(1) The addition of salt significantly reduced the dissolution of the CH_4_ + CO_2_ mixture in water. For the solubility of pure gas in water, the prediction result of the PR-EOS solubility model was the best. However, for the water solubility prediction of the CH_4_+ CO_2_ mixture, the prediction accuracy of the SRK-EOS solubility model was better.
(2) During gas invasion, the invaded gas containing CO_2_ and H_2_S exists in the wellbore in a gaseous state. Dissolution under low invasion (0.9 m^3^) leads to a change in the volume fraction of free gas and an increase in concealment. All the invaded gas was dissolved in the drilling fluid before reaching its saturation state. After the saturation state, the gas was separated out, the volume of free gas increased sharply. The closer the separation expansion was to the wellhead, the more sudden was the swelling in the later stage of migration.
(3) The higher the contents of CO_2_ and H_2_S in the invaded gas, the greater is the dissolution in the drilling fluid; the smaller the volume fraction of the free gas phase in the wellbore, the farther is the gas front from the wellhead. The gas dissolution effect increased the time at which the mud pit gain reached a warning value. Consequently, the higher the acid gas content, the stronger is the concealment during gas invasion in low-permeability reservoirs.

## APPENDIX A

For the viscosity calculation of sour natural gas, the relationship of Carr et al. (1954) is adopted^26^

A1

Among them, the values of coefficients *A*_0_*–A*_15_ are listed in Table A1.

A2

where, μ_H2S_, μ_N2_, andμ_CO2_ are the viscosity correction value of H_2_S, N_2_, and CO_2_ respectively.

A3

A4

A5

A6

where, μ is the viscosity, MPa·s. *T* is the temperature, °C.

**Table A1:** Values of Coefficients *A*_0_*–A*_15_

*A*_0_	–2.4621182	*A*_8_	–0.793385684
***A***_**1**_	2.97054714	*A*_9_	1.39643306
***A***_**2**_	–0.286264054	*A*_10_	–0.149144925
***A***_**3**_	0.00805420522	*A*_11_	0.00441015512
***A***_**4**_	2.80860949	*A*_12_	0.0839387178
***A***_**5**_	–3.49803305	*A*_13_	–0.186408848
***A***_**6**_	0.36037302	*A*_14_	0.0203367881
***A***_**7**_	–0.0104432413	*A*_15_	–0.000609579263

## APPENDIX B

For the calculation of the liquid-phase activity coefficient, if it is pure aqueous solution, it is considered that the dissolution of gas component *i* in aqueous solution is very small, γ_*i*_ = 1. In the case of electrolyte solution, the activity coefficient of gas component *i* adopts the calculation equation of Duan and Sun:^40^

B1

where *m*_c_ and *m*_a_ represent the molar mass fractions of cations and anions, respectively. λ and ξ are the second-order and third-order interaction parameters depending on temperature and pressure, and the second-order interaction parameters of gas and anion λ_i–a_ is assumed to be 0.^23,24,40^ Parameter values λ_i–a_ and ξ_i–a–c_ are solved by the following formula, and the parameter values are listed in Table B1.

B2

**Table B1:** Parameter Values of Equation (B2)

coefficient						
c_1_	–0.0652869	–1.144624 × 10^–2^	1.03658689	–0.010274152	–5.7066455 × 10^–1^	–2.9990084 × 10^–3^
c_2_	1.6790636 × 10^–4^	2.8274958 × 10^–5^	–1.1784791 × 10^–3^	0	7.2997588 × 10^–4^	0
c_3_	40.838951	0	–1.7754826 × 10^2^	0	1.52 × 10^2^	0
c_4_	0	0	–4.5313285 × 10^–4^	0	3.1927112 × 10^–5^	0
c_5_	0	0	0	0	0	0
c_6_	–3.9266518 × 10^–2^	1.3980876 × 10^–2^	0	0	–1.6426510 × 10^–5^	0
c_7_	0	0	0	0	0	0
c_8_	2.1157167 × 10^–2^	–1.4349005 × 10^–2^	0	0	0	0
c_9_	6.5486487 × 10^–6^	0	0	0	0	0
c_10_	0	0	0.47751650 × 102	0	0	0
