# Effects of Gas Dissolution on Gas Migration during Gas Invasion in Drilling

**DOI:** 10.1021/acsomega.2c07384

**Published:** 2022-12-04

**Authors:** Haikang He, Baojiang Sun, Xiaohui Sun, Zhiyuan Wang, Xuefeng Li

There was an omission in the acknowledgment in the original manuscript. The corrected acknowledgment is given below.

The authors sincerely thank the Postdoctoral innovative talents support program in China (BX2021374) and the National Natural Science Foundation--Youth Foundation (5210040269) for providing financial support, and Key Supporting Projects of Joint Fund of National Natural Science Foundation of China (U21B2069).

